# Integrating Moisture Sorption, Hygroscopic Kinetics, and Mechanical Analysis to Forecast Leakage and Shelf Life of Gelatin Soft Capsules

**DOI:** 10.3390/gels12030213

**Published:** 2026-03-05

**Authors:** Siyu Pan, Chao Xie, Chungang Zhang, Zitong Qiao

**Affiliations:** College of Pharmacy, Liaoning University of Traditional Chinese Medicine, Dalian 116600, China; psy18242960598@163.com (S.P.); xiechaojy1019@163.com (C.X.); 19818805106@163.com (Z.Q.)

**Keywords:** gelatin, soft capsules, leakage, stability, Arrhenius equation, the generalized Eyring model

## Abstract

The objective of this study was to develop a predictive methodology for assessing the leakage phenomenon of gelatin-based soft capsules under various storage conditions. The equilibrium moisture content of the soft capsules was influenced by the temperature and humidity. The leakage phenomenon was attributable to the swelling of gelatin, as revealed by Fourier Transform Infrared spectroscopy (FT-IR) and Scanning Electron Microscopy (SEM) imaging techniques. Additionally, the moisture diffusion mechanism of soft capsule shells was systematically investigated based on the principles of hygroscopic kinetics, enabling quantitative evaluation of their hygroscopic performance under different environmental conditions. Based on macromechanical analysis, the mechanical failure curves of soft capsule shells under different environmental conditions were investigated, enabling successful determination of the shelf life of the soft capsules. Importantly, the Arrhenius equation and the generalized Eyring model were introduced to successfully predict the occurrence of leakage during storage. The developed prediction method performs successful and accurate stability assessment under various conditions, which is crucial for the development of soft capsules.

## 1. Introduction

Soft capsules have gained significant attention in pharmaceutical applications owing to their distinct advantages, including ease of administration, improved stability, and the ability to encapsulate liquids [1]. Of the current preparation methods for soft capsules, the one that is mainly used involves manufacturing through the rotary die method. This method has led to gelatin becoming the primary material for the shell of soft capsules. Gelatin is a protein derived from the skin and bones of animals that contains 18 different amino acids. It is precisely because of its unique properties, such as biodegradability and safety, that gelatin is widely used in the food and pharmaceutical industries [2]. Nevertheless, despite these advantages, gelatin-based soft capsules present formulation challenges, particularly in terms of physicochemical stability during storage and transportation. Content leakage, the primary manifestation of stability issues in gelatin-based soft capsules during storage, has always been a challenging problem. The underlying cause of this phenomenon can be attributed to the fact that the soft capsules are particularly influenced by external conditions. For example, ultraviolet (UV) radiation from sunlight may cause denaturation and aggregation of the micelles in the capsule shell, which affects the mechanical properties and significantly increases the risk of content leakage [3]. When the drug comes into contact with light, it may give rise to the changes in the molecular structure of the drug, leading to the decomposition or loss of effectiveness of the active ingredients. At the same time, the oxygen in the air may react with the capsule shell material through oxidation, leading to a reduction in the mechanical strength of the shell. This situation can result in potential cracking or leakage, thereby compromising the stability and efficacy of the drug [4]. Additionally, microorganisms in the air, such as bacteria and molds, may adhere to the surface of the capsule shell and generate chemical substances that can chemically react with the shell. This change will reduce the stability of the capsule, ultimately leading to leakage phenomena [3]. Furthermore, the presence of hydrophilic groups in the gelatin and glycerol molecules confers substantial hygroscopic characteristics to the soft capsules under high humidity conditions [5,6,7]. When excessive moisture enters the capsule shell, it can lead to the deterioration of the mechanical properties of capsule shells, increasing the likelihood of problems such as adhesion and even leakage [8]. However, this issue becomes further exacerbated with a change in temperature. As the temperature increases, the mobility of molecular chains is enhanced. Thus, the intermolecular spaces expand, providing more channels and pathways for the penetration of water molecules [9]. Among all the aforementioned factors, temperature and humidity are the most critical and prevalent elements influencing the mechanical properties of soft capsules, ultimately contributing to leakage issues. The frequent occurrence of leakage during soft capsule formulation cannot be ignored. Therefore, if the leakage phenomenon of soft capsules can be predicted, it will provide guidance for evaluating the stability of soft capsules, which holds significant importance for the development of soft capsule formulations. So, what methods can we use to predict the occurrence of leakage phenomenon of capsule shells?

Currently, the plotting of moisture sorption isotherms is a common method to describe the moisture absorption behavior of soft capsules by determining equilibrium moisture content [8]. In addition, many researchers have determined the rate of mass change in materials after exposure to moisture for varying durations and plotted curves to compare the moisture absorption capabilities of different materials [3,10,11]. However, mathematical models able to more accurately evaluate the moisture absorption properties of materials have yet been not found. Moreover, mechanical properties have not been incorporated into experiments assessing the stability of capsule shells, nor has the combination of mechanical properties with mathematical models been utilized to achieve a precise evaluation.

The soft capsule shell is essentially a typical physical gel, consisting of a three-dimensional network formed by gelatin polymers through physical cross-linking (primarily hydrogen-bonded triple-helix-like structures), with water and plasticizers filling the network. During storage, gelatin capsule shells are exposed to various humidity conditions; water molecules, acting as plasticizers, penetrate the gel network and induce network swelling. Therefore, the prediction of soft capsule shelf life is fundamentally the prediction of the failure time of the gelatin physical gel network as it evolves over time. From the perspective of gel science, this study monitors the hygroscopicity and mechanical properties of the gel network during moisture absorption, and introduces the Arrhenius equation and Eyring model to establish a failure prediction model for the gelatin gel network, thereby providing a theoretical basis for the stability evaluation of soft capsule preparations.

The Huoxiang Zhengqi Soft Capsule is composed of the contents and capsule shell materials. The contents include *Atractylodes macrocephala* (Cangzhu), *Citri Reticulatae Pericarpium* (Chenpi), *Magnolia officinalis* (Houpo), *Angelicae Dahuricae Radix* (Baizhi), *Poria* (Fuling), *Arecae Pericarpium* (Dafupi), raw *Pinelliae Rhizoma* (Banxia), *Glycyrrhizae* (Gancao), *Pogostemon cablin* (Guanghuoxiang), and *Perilla frutescens* (Zisuye). It has the effects of relieving exterior syndrome and resolving dampness, regulating qi and harmonizing the middle-jiao [12]. The Huoxiang Zhengqi formula originates from the Tai Ping Hui Min He Ji Ju Fang (Formulary of the Peaceful Benevolent Dispensary) of the Song Dynasty. It is a classic prescription for treating external contraction of wind-cold and internal damage of dampness. Huoxiang Zhengqi Soft Capsules, as the modern dosage form of this formula, represent the first traditional Chinese medicine (TCM) compound developed in a soft capsule formulation nationwide. This represents an important step in the history of traditional Chinese medicine (TCM) soft capsule development. Studies have confirmed that Huoxiang Zhengqi Soft Capsules possess definite pharmacological activities, including protective effects on intestinal barrier function, therapeutic mechanisms in functional dyspepsia, and regulatory effects on diarrhea-predominant irritable bowel syndrome [13,14]. Therefore, this product was selected as the research object to provide a reference for the quality control of similar TCM soft capsules. The capsule shell material is primarily composed of gelatin; therefore, Huoxiang Zhengqi Soft Capsules should be classified as gelatin-based soft capsules. This experiment used Huoxiang Zhengqi soft capsules as a model drug, primarily focused on the study of the soft capsule shells. Although plant-based and polymer materials have demonstrated certain advantages in terms of performance in recent years, their high production costs have limited their widespread adoption in Chinese pharmaceutical enterprises at the current stage [15]. In contrast, gelatin derived from animal skin and bone remains the mainstream material for soft capsule production. However, gelatin shells are susceptible to aging and cross-linking during storage due to factors such as temperature, humidity, and fill migration—this existing problem is precisely the core concern of this study [16]. Therefore, based on the actual circumstances of China’s pharmaceutical industry, selecting gelatin soft capsules as the research object carries clear practical significance.

The soft capsule shell is essentially a typical physical gel, consisting of a three-dimensional network formed by gelatin polymers through physical cross-linking (primarily hydrogen-bonded triple-helix-like structures), with water and plasticizers filling the network [1,6]. During storage, gelatin capsule shells are exposed to various humidity conditions; water molecules, acting as plasticizers, penetrate the gel network and induce network swelling.

This paper aimed to predict leakage stability based on hygroscopic kinetics and macromechanics. Moisture sorption isotherms were constructed to investigate how environmental temperature and humidity affect the hygroscopicity of the capsule shells. The changes in the internal functional groups and microscopic morphology of gelatin during the leakage process were analyzed by Fourier Transform Infrared (FT-IR) spectroscopy and Scanning Electron Microscopy (SEM), which revealed the effect of the moisture on the capsule shells. Based on moisture absorption kinetics and macroscopic mechanics, the moisture diffusion coefficient and shelf life were evaluated, directly reflecting the leakage stability of the capsule shell. In addition, the introduction of the mathematical models successfully predicted the probability of leakage phenomena and when it would occur in the capsule shell. The establishment of two models provides conceptual guidance for the evaluation methods of soft capsule stability during storage. Therefore, the prediction of soft capsule shelf life was fundamentally the prediction of the failure time of the gelatin physical gel network as it evolves over time. From the perspective of gel science, this study monitored the hygroscopicity and mechanical properties of the gel network during moisture absorption, and introduced the Arrhenius equation and Eyring model to establish a failure prediction model for the gelatin gel network, thereby providing a theoretical basis for the stability evaluation of soft capsule preparations.

## 2. Results and Discussion

### 2.1. Moisture Sorption Isotherms of Soft Capsules

**Figure 1:**
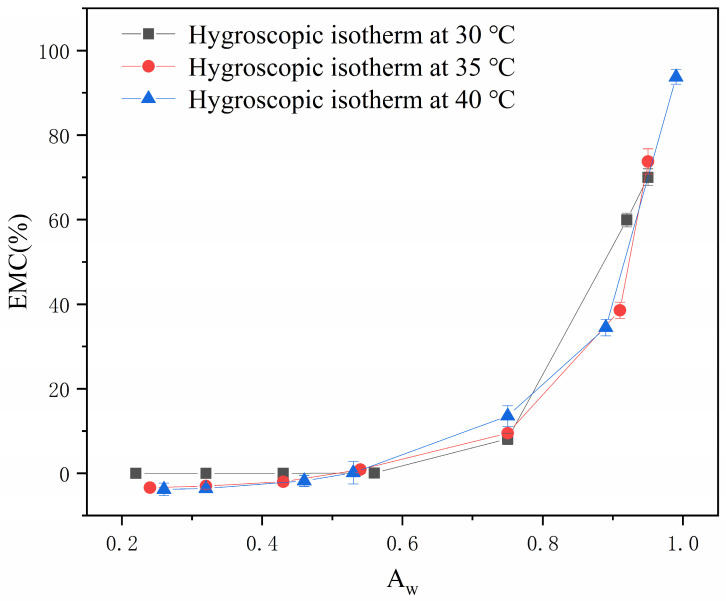
Hygroscopic isotherm of the soft capsules.

Figure 1 illustrates the moisture isotherms of the soft capsules under different temperature and humidity conditions, with their hygroscopic behavior exhibiting significant stage-specific characteristics. When the water activity was within 0.4, the EMC of the capsule shell remained relatively stable without significant changes. This sorption pattern can be attributed to the monomolecular layer adsorption of hydrophilic functional groups (such as hydroxyl, carboxyl, and amino groups) in the gelatin molecular chains being close to saturation [17]. However, when water activity exceeded 0.6, the EMC showed a sharp upward trend with increasing water activity, a phenomenon consistent with the capillary condensation theory and multi-layer adsorption mechanism [18]. This hygroscopic pattern is highly consistent with the characteristics of Type II isotherms (S-shaped curves) in the IUPAC classification. According to Brunauer’s classification criteria, the fitting results of the actual data in Table 1 using the GAB model indicated that under all temperature conditions, when the fitting parameters satisfied C > 2 and k approached 1, they consistently conformed to this classification standard [19]. This situation further verified that the hygroscopic behavior conforms to Type II isotherm characteristics, indicating that the strong interaction between the gelatin matrix and water molecules dominates the multi-layer adsorption process. Furthermore, a positive correlation between EMC and temperature was observed. The application of heat enhanced molecular chain mobility within the gelatin matrix, leading to weakening intermolecular forces and subsequent expansion of the polymer network. These structural changes facilitated water molecule penetration into the gelatin structure, ultimately contributing to capsule instability. Therefore, these findings demonstrated that the leakage phenomenon in soft capsules was influenced by both temperature and humidity conditions during storage.

### 2.2. FT-IR Analysis of Soft Capsules

**Figure 2:**
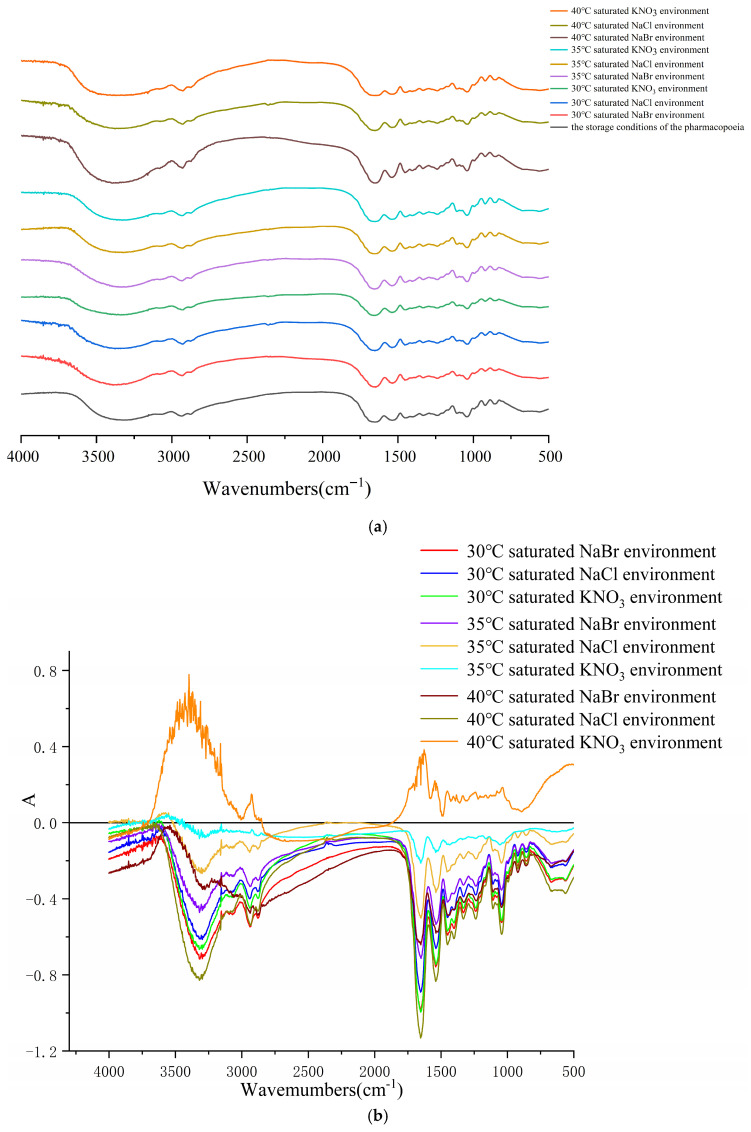

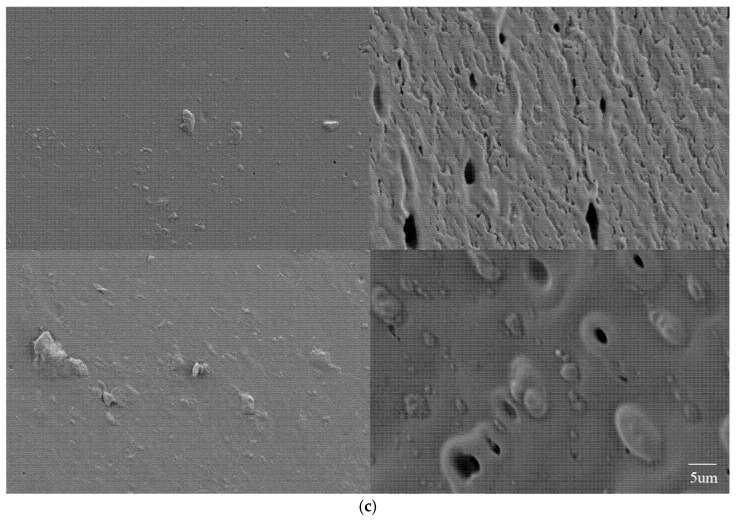
(**a**) FT-IR spectra of the soft capsules under different storage conditions. (**b**) Difference spectrum of the soft capsules under different storage conditions. (**c**) The appearance and cross-sectional morphology of the soft capsules before and after moisture absorption (Top: Before moisture absorption; Bottom: After moisture absorption; Left: Appearance; Right: Cross-section).

Figure 2a presents the FT-IR spectral analysis of soft capsules under various storage conditions. The band corresponding to the amide A band at 3288 cm^−1^ was mainly attributed to the vibration of the -NH and -OH groups in gelatin molecules [20]. A weak absorption observed to appear at 2933 cm^−1^ was attributed to the vibration of the C-N band within the amide B band. The absorption bands at 1633 cm^−1^ originated from the C=O absorption band of amide I in the gelatin structure [21]. The band at 1547 cm^−1^ was related to the deformation vibrations of N–H groups and the stretching vibrations of C–N groups in the amide II band. The amide III band, situated at approximately 1239 cm^−1^, was produced by νC-N and δN-H vibrations [22]. The spectra of the soft capsule shells showed overall similarity before and after moisture absorption, but notable differences were observed in specific bands.

Dynamic changes in functional groups between samples were precisely characterized by infrared difference spectrum analysis. As shown in Figure 2b, the difference spectrum between the stored sample and the initial sample exhibited significant negative bands at 3300, 1650 and 1550 cm^−1^, indicating that the amide bonds (-CONH-) in gelatin molecules were broken, leading to a reduction in the number of N-H and C=O groups. This phenomenon was attributed to the hydrolysis reaction of gelatin amide bonds (C-N) induced by a high-humidity environment. This process led to a decrease in the N-H and C=O functional groups, consequently resulting in the appearance of negative bands. However, the formation of positive bands appeared when soft capsules were put under extreme conditions (40 °C saturated KNO_3_ environment), which can be attributed to the thermal degradation of gelatin, generating new hydrogen-containing functional groups. These experimental results were similar to the findings of Berthomieu et al. [23]. This approach of utilizing infrared difference spectroscopy to capture subtle structural changes in proteins/polypeptides under external stimuli has previously been successfully applied to investigate the conformational dynamics of complex biomolecules. For instance, Berthomieu et al. employed electrochemically induced FTIR difference spectroscopy to conduct an in-depth analysis of minor changes in metal–ligand interactions within redox proteins. These results further confirmed that environmental humidity and temperature were key factors contributing to the deterioration of soft capsule stability.

### 2.3. SEM Analysis of Soft Capsules

Figure 2b illustrates the appearance and cross-sectional morphology of the soft capsules before and after moisture absorption. The shell of the soft capsules exhibited a relatively smooth structure on the surface, while the cross-section appeared comparatively rough. By comparing the cross-sections before and after moisture absorption, it was evident that the cross-section became smoother after moisture absorption, and the porosity of soft capsules demonstrated a significant increase. The swelling ratio of the hydrated soft capsule shell was determined to be 62.50 ± 12.15%. This was explained by the swelling effect of gelatin, resulting from water penetration and subsequent volume expansion of the molecules. The experimental results based on microscopic morphology images further revealed the underlying essence of the leakage phenomenon. Moisture penetration was the fundamental cause of capsule rupture and leakage. Initially, water infiltrated the disordered regions of the capsule shell, disrupting the internal hydrogen bonding network and reducing the material’s strength. As moisture accumulated locally, uneven swelling generated internal stress within the shell. Under minor external forces, microcracks gradually formed at weak points. Once these cracks propagated to a critical extent, they ultimately led to complete rupture or leakage.

### 2.4. Hygroscopic Weight Gain Experiment of Soft Capsules

#### 2.4.1. Determination of the Rate of Change in Hygroscopic Mass of Soft Capsules

**Figure 3:**
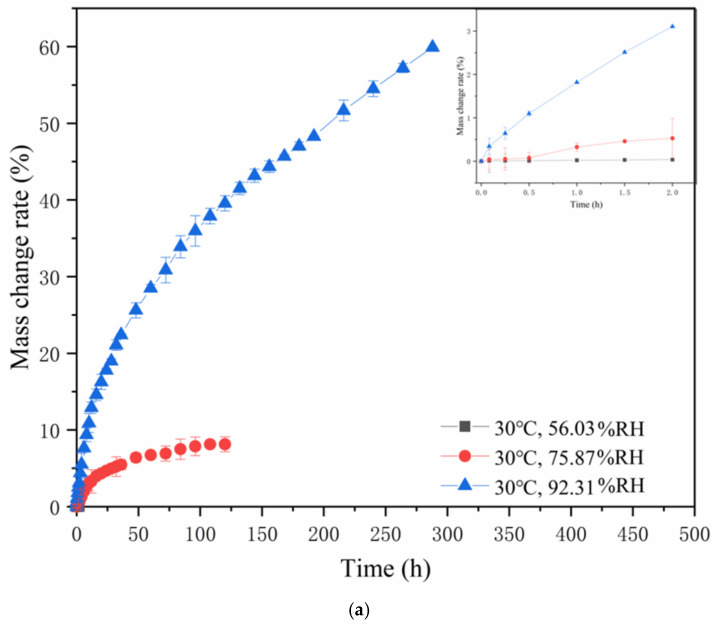

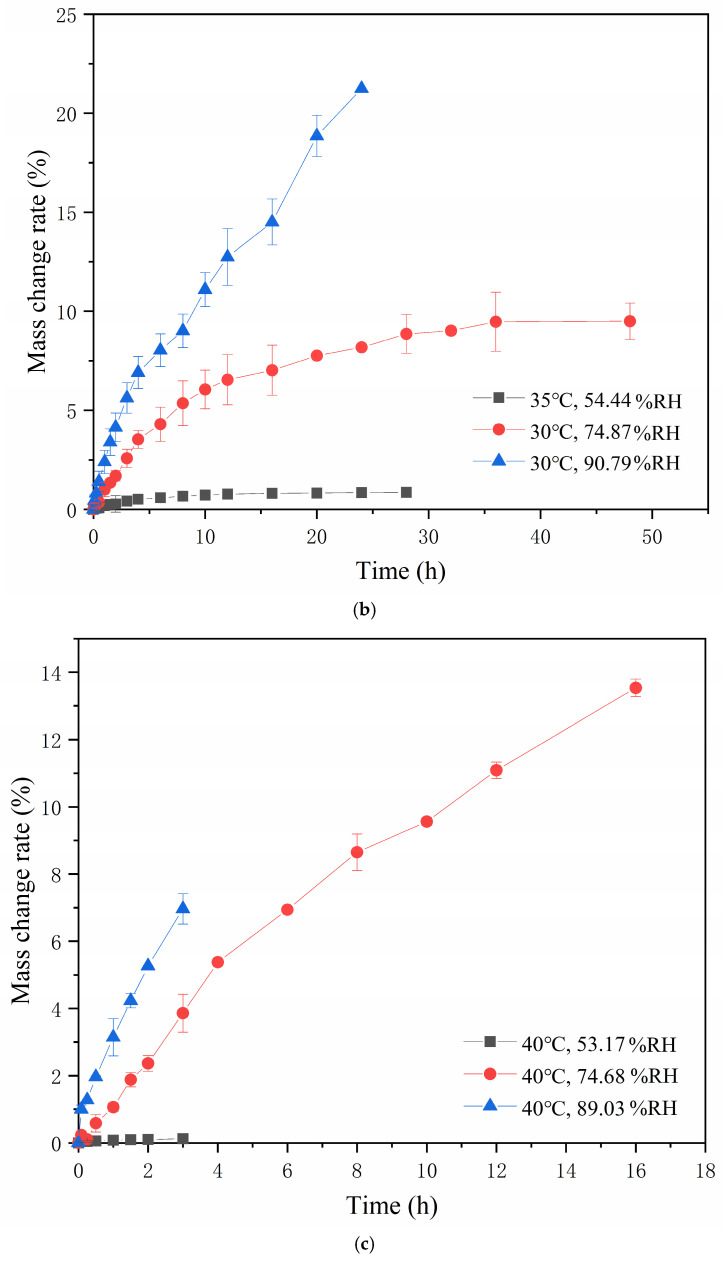
The mass change rate curves of soft capsules: (**a**) Mass change rate curve at 30 °C; (**b**) Mass change rate curve at 35 °C; (**c**) Mass change rate curve at 40 °C.

According to Figure 3, a slight decrease in the mass variation rate may be observed during the plateau phase. This phenomenon was likely associated with the evolution of hydrogen bonding interactions between water molecules and the polar sites in the hygroscopic material. As water molecules permeated the system, the hydrogen bonding transitioned from an initially strong and tightly bound state to a progressively weaker and more dissociated state. Moreover, the mechanism of the exothermic reaction during the moisture adsorption process also contributed to the slight decrease in the mass of change rate. Additionally, the exothermic nature of the moisture adsorption process may contribute to the observed reduction in the mass of change rate. The reason was that the release of thermal energy could influence the equilibrium dynamics of the system [24,25]. The diffusion behavior of water molecules during moisture absorption of gelatin capsule shells was significantly influenced by the evolution of their gel network structure. In the initial stage of moisture absorption, water molecules entered the network, causing swelling, enhanced chain segment mobility, and enlarged network pores. Diffusion followed Fickian behavior, manifested as a rapid rise in the moisture absorption curve. With prolonged moisture absorption time, structural changes occurred between gelatin molecular chains, leading to increased diffusion resistance, and the diffusion behavior transitioned to non-Fickian behavior. At this stage, the relaxation of network chains became the rate-limiting step, manifested as a plateau in the moisture absorption curve. The FT-IR results of this study confirmed structural changes within the gelatin molecules, and the SEM images revealed the evolution of the network microstructure (from dense to swollen). The two-stage characteristic of the hygroscopic kinetics curve was consistent with the above mechanism. Therefore, the evolution of the gel state, by altering diffusion pathways and kinetics, ultimately affected the stability and shelf life of the capsule shell.

#### 2.4.2. Diffusion Behavior Analysis of Soft Capsules

The experimental data in Figure 3 was analyzed using OriginPro 2019b statistical analysis software for nonlinear curve fitting according to Equation (4). The diffusion kinetic parameters (n) of soft capsules under different conditions is shown in Table 2. According to Table 2, the *n*-values all fall near 0.5, which indicates that the moisture absorption behaviors of all three films conform to the Fickian diffusion model [26]. In other words, the moisture diffusion mechanism essentially follows a simple passive diffusion process where water molecules migrate from areas of higher concentration to areas of lower concentration.

#### 2.4.3. Establishment of k′ of Soft Capsules

The Fickian diffusion was a suitable framework for modeling the water absorption results. According to Equation (5), the slope k′ can be calculated; the results are shown in Table 3.

#### 2.4.4. The Fitting of the Arrhenius Formula of Soft Capsules

**Figure 4:**
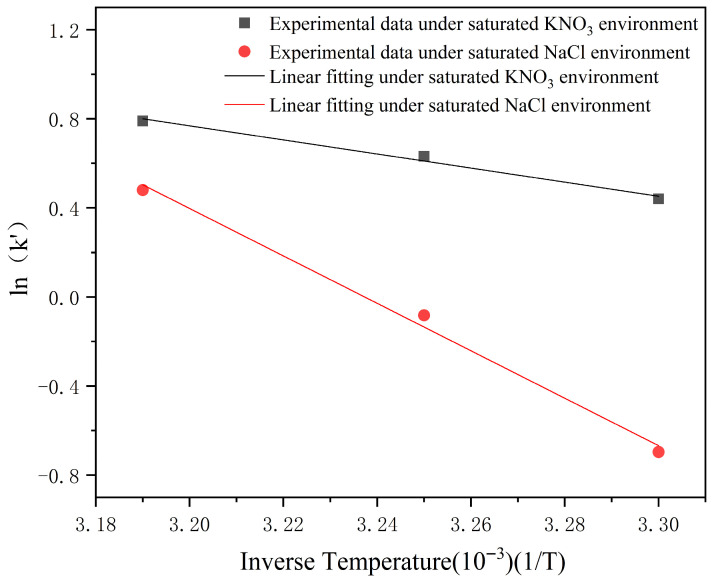
Arrhenius curve of the soft capsules.

The hygroscopic effect of the soft capsules was relatively insignificant in a saturated NaBr environment. In this study, the Arrhenius curves were exclusively fitted for the soft capsules under saturated NaCl and KNO_3_ environments. According to Equation (8), the Arrhenius curves were successfully fitted, as shown in Figure 4. The fitting results were as follows:

ln k′(NaCl) = 34.47657 − 10.64886 × (1/T), R^2^ = 0.988;

ln k′(KNO_3_) = 10.88146 − 3.16038 × (1/T), R^2^ = 0.977.

**Figure 5:**
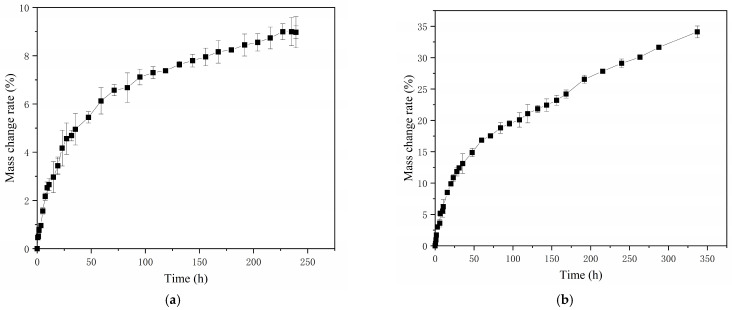
The mass change rate curves of the soft capsules: (**a**) Mass change rate curve under saturated NaCl humidity at 25 °C; (**b**) Mass change rate curve under saturated KNO_3_ environment at 25 °C.

To ensure the predicted equation reliability, the moisture absorption coefficient at 25 °C under saturated NaCl and KNO_3_ environments was predicted and compared with the actual moisture absorption coefficient. Based on the fitted formula, the predicted k′ in a saturated NaCl environment at 25 °C was calculated to be 0.28942, while the actual value was 0.27754. The predicted k′ was 1.32396 in a saturated KNO_3_ environment at 25 °C, and the experimentally determined k′ was also 1.32396, as shown in Figure 5. And the relative error between the predicted and experimental values was 4.28% in the saturated NaCl environment and 0.01% in the saturated KNO_3_ environment. These results effectively demonstrated that the Arrhenius equation can successfully predict the moisture absorption capacity of soft capsule shells under different environmental conditions.

### 2.5. Effect of Moisture on Mechanical Properties of Soft Capsules

#### 2.5.1. Fitting of the Curve and Determination of Failure Time of Soft Capsules

**Figure 6:**
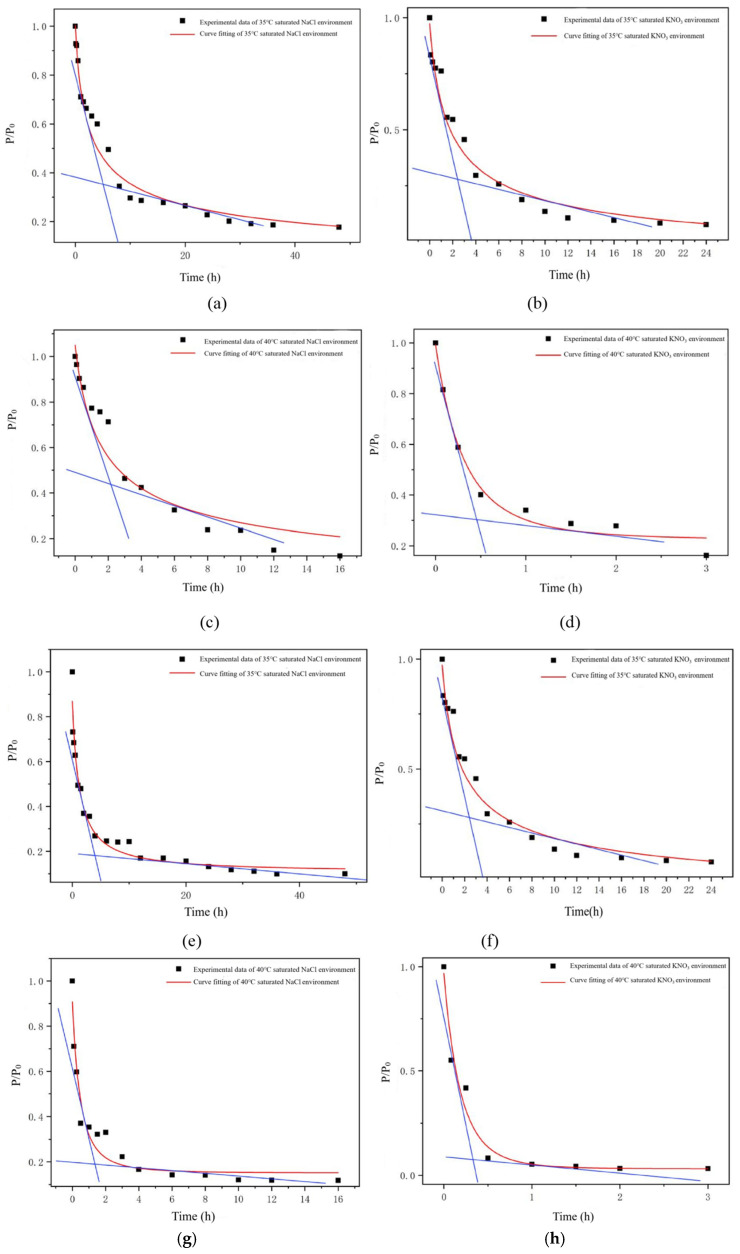
Curve fitting of puncture load of the soft capsules: (**a**) Curve fitting of puncture load under saturated NaCl humidity at 35 °C; (**b**) Curve fitting of puncture load under saturated KNO_3_ humidity at 35 °C; (**c**) Curve fitting of puncture load under saturated NaCl humidity at 40 °C; (**d**) curve fitting of puncture load under saturated KNO_3_ humidity at 40 °C. Curve fitting of tensile load of the soft capsules: (**e**) Curve fitting of tensile load under saturated NaCl humidity at 35 °C; (**f**) Curve fitting of tensile load under saturated KNO_3_ humidity at 35 °C; (**g**) Curve fitting of tensile load under saturated NaCl humidity at 40 °C; (**h**) curve fitting of tensile load under saturated KNO_3_ humidity at 40 °C. The blue line is drawn as the tangent at the inflection point of the curve.

In accordance with Equation (9), the curves depicting the variation in the mechanical properties of soft capsules over time in different environments were plotted, as shown in Figure 6. Water existed as a plasticizer within the gelatin system. The hydrogen bonds were formed between functional groups of the plasticizer and the gelatin molecules, weakening the intermolecular forces between the gelatin molecules. Thus, both the puncture and tensile loads exhibited a gradual decrease trend. Through the analysis of these curves, the shelf life of the soft capsules was calculated and determined, as shown in Table 4.

#### 2.5.2. Prediction of Storage Time of Soft Capsules

Table 2 presents the shelf life of soft capsules in the puncture test under various environmental conditions. Substituted into Equation (11), the prediction model for shelf life in the puncture experiment was derived as follows:

(1) $$\ln\xi T=214.60-6647.80\varphi_{1}(T)-355.10\varphi_{2}(H)+107,812.02\varphi_{1}(T)\varphi_{2}(H)$$

**Figure 7:**
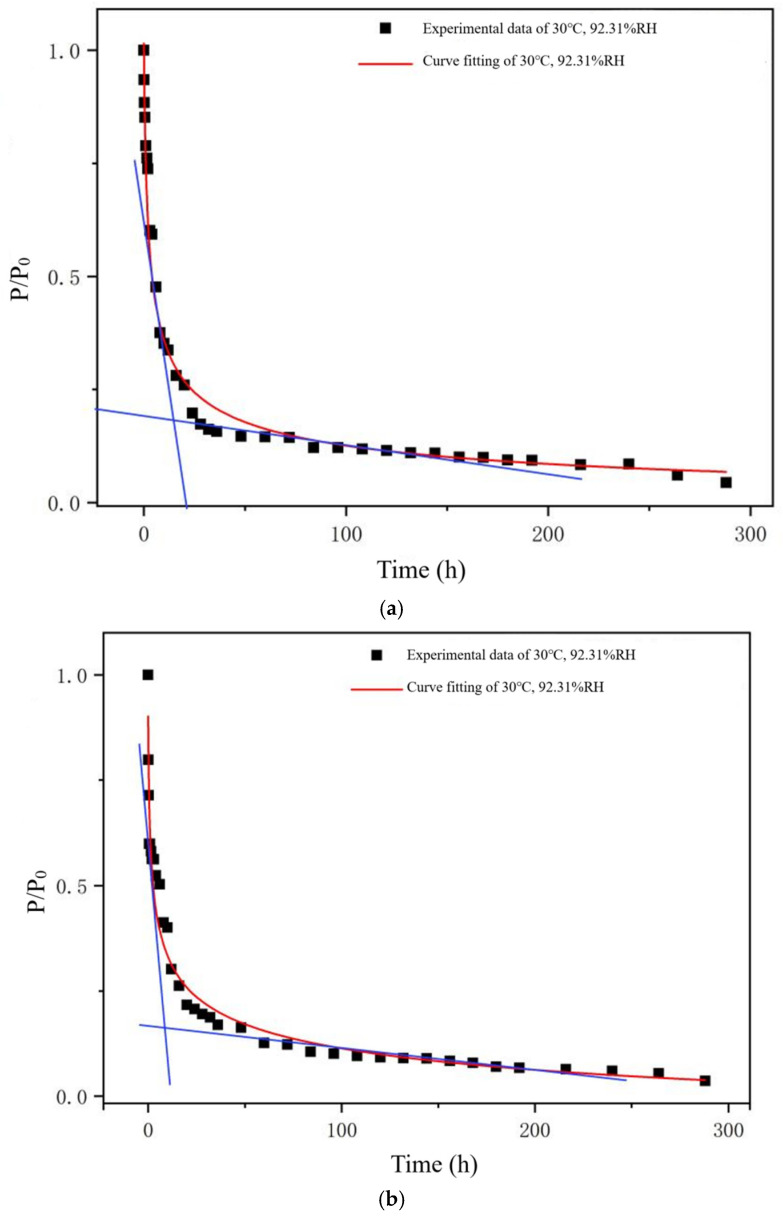
(**a**) Puncture load of soft capsules at 30 °C, 92.31% RH. (**b**) Tensile load of soft capsules at 30 °C, 92.31% RH. The blue line is drawn as the tangent at the inflection point of the curve.

The experimental data and fitting curve in the puncture experiment at 30 °C and 92.31% RH are illustrated in Figure 7a. The actual shelf life of soft capsules in the puncture test in a saturated KNO_3_ environment at 30 °C was 16.10 h. Under the same condition, the shelf life of soft capsules was calculated to be 16.22 h through prediction.

Table 2 presents the shelf life of soft capsules in the tensile test under different environmental conditions. Substituted into Equation (10), the prediction model for the shelf life in the tensile experiment was derived as follows:

(2) $$\ln\xi T=91.83-24,450.02\varphi_{1}(T)-203.03\varphi_{2}(H)+60,346.16\varphi_{1}(T)\varphi_{2}(H)$$

The experimental data and fitting curve in the tensile experiment in saturated KNO_3_ environment at 30 °C was illustrated in Figure 7b. The actual shelf life of soft capsules in the tensile test in saturated KNO_3_ environment at 30 °C was 6.72 h. Under the same condition, the shelf life of soft capsules was calculated to be 6.40 h through prediction.

To verify the reliability of the model, the relative error between the experimental and predicted values was calculated. Specifically, the relative error between the actual shelf life in the puncture test and the predicted value was 0.74%, while that for the tensile test was 4.76%. This demonstrated that the predictions of the model were accurate.

It should be noted that the quantitative prediction model of this framework was established based on the characteristic parameters of gelatin materials. For non-gelatin or composite shell materials such as hydroxypropyl methylcellulose (HPMC) and pullulan, due to their fundamental differences in hydrophilicity, thermodynamic properties, and structural characteristics, the key transport and deformation parameters within the framework required recalibration. The application of this method will offer a reference approach aiming to extend it to a broader range of material systems in the future, thereby enhancing its value as a universal tool in the pharmaceutical industry.

## 3. Conclusions

In this study, the leakage stability of gelatin-based soft capsules has been evaluated and predicted. The adsorption isotherms demonstrated that the equilibrium moisture content was dependent on both temperature and humidity, increasing as either parameter increased. The internal and external structures of the capsule shell before and after moisture absorption, as revealed by FT-IR and SEM, were analyzed. The ingress of moisture led to changes in the functional groups of gelatin molecules and the external microstructure. Based on the moisture adsorption kinetics, the curve of the mass change rate was plotted as a function of time to evaluate the moisture absorption capacity of gelatin-based soft capsule shells under different storage environments. In addition, the principles from macromechanics were introduced to establish a curve showing the variation in mechanical indicators over time, thereby enabling the calculation of the capsule shell. Furthermore, the two predictive models were developed to determine the leakage stability of gelatin-based soft capsule shells during the storage process. The experimental results indicated that the models exhibited perfect fitting performance and could serve as a valuable tool for predicting the moisture absorption capacity and shelf life of soft capsules. This mathematical model primarily focused on the influence of temperature and humidity, while other factors were not taken into consideration. There are many factors, such as light, oxygen, and microorganisms like bacteria, which played a dominant role in causing leakage phenomena during storage. However, in practical terms, current packaging methods such as aluminum foil pouch sealing can mitigate the adverse effects caused by light exposure. As for the factor of oxygen, its impact on disintegration time was significantly greater than on mechanical properties. When microorganisms affected the stability of capsules, drug deterioration became the primary concern, rendering the assessment of stability somewhat meaningless. Therefore, despite certain limitations, this mathematical model remained applicable for predicting leakage phenomena. Currently, the traditional methods for evaluating shelf life in stability tests according to the Chinese Pharmacopeia include accelerated testing and long-term testing, which typically require six months to a year to yield experimental results. However, the shelf life of soft capsules can be evaluated in just a few days of experimentation with the model established in this study. The development of this mathematical model significantly reduced experimental time while accurately predicting shelf life. The introduction of these models provided theoretical guidance for evaluating the stability of soft capsules and held significant importance for the research and development of soft capsule formulations.

## 4. Materials and Methods

### 4.1. Materials

Huoxiang Zhengqi Soft Capsules (typically oval or oblong in shape, smooth and soft outer shell, dark brown in color) were obtained from Shenwei Pharmaceutical Group Co., Ltd., Shijiazhuang, China. CH_3_COOK, MgCl_2_, K_2_CO_3_, NaBr, NaCl and KNO_3_ were purchased from Xilong Scientific Co., Ltd., Guangdong, China. Purified water (H_2_O; Wahaha Group Co., Ltd., Hangzhou, China) was used to prepare all solutions.

### 4.2. Determination of Equilibrium Moisture Content of Soft Capsules

To systematically evaluate the hygroscopic behavior of soft capsule shells under different temperature and humidity conditions, the saturated salt solution method was employed to create constant relative humidity environments in sealed containers. Six salts (CH_3_COOK, MgCl_2_, K_2_CO_3_, NaBr, NaCl and KNO_3_) were selected to generate relative humidity levels covering a relatively wide range. All salt solutions were prepared at room temperature, with excess solute added to ensure that the solutions remained saturated throughout the entire experimental period. The prepared saturated salt solutions were placed at the bottom of glass desiccators, which were then transferred into constant temperature incubators set at 30 °C, 35 °C and 40 °C, respectively. According to the ASTM E104 standard, each salt solution corresponds to specific equilibrium relative humidity values at different temperatures. To ensure the accuracy of the experimental conditions, the actual temperature and humidity inside each desiccator were verified using a calibrated hygrometer (Shenzhen Jumao Yuan Technology Co., Ltd., Shenzhen, China) before sample placement. The hygroscopic experiment was initiated only after confirming that the deviations between the measured values and the ASTM E104 standard [27] reference values were within an acceptable range. The actual humidity values are detailed in Table 5. The soft capsules were selected and placed in a specific environment. The weight of the soft capsules in the environment was recorded using an analytical balance with a precision of 0.01 mg (Tianjin Tianma Hengji Instrument Co., Ltd., Tianjin, China) until equilibrium was reached, defined as a weight change of less than 0.01%. The equilibrium moisture content (EMC) was calculated using the following equation:

(3) $$EMC=\frac{\left(W_{\infty }-W_{0}\right)}{W_{0}}\times 100\%$$

where *W_∞_* is the equilibrium moisture content; *W_0_* is the moisture content at initial time. Experimental data were modeled using the GAB equation:

(4) $$EMC=X_{0}CAw/(1-KA_{w})(1-KA_{w}+KCA_{w})$$

where *K* and *C* are the thermodynamic constants of the GAB model and *X_0_* is the monolayer water content of the soft capsule.

### 4.3. FT-IR Analysis of Soft Capsules

FT-IR spectroscopy was utilized to characterize structural modifications in soft capsules following moisture exposure under varying environmental conditions. The soft capsules were stored at different temperature and relative humidity combinations for 20 days. Then, the soft capsules were taken out, the content was removed, and the shells were thoroughly cleaned to ensure analytical accuracy. The structural characterization was performed on a FTIR-850 infrared spectrometer (Tianjin Port East Technology, Co., Ltd., Tianjin, China). FT-IR spectra were recorded at the wave number range of 400–4000 cm^−1^, with a resolution of 4 cm^−1^ and an average of 32 cumulative scans.

### 4.4. SEM Analysis of Soft Capsules

The soft capsules were stored in a saturated NaCl environment at 25 °C to allow for moisture absorption. Then, the soft capsules were taken out after 24 h, the content was removed, and the shells were thoroughly cleaned. The surface and cross-sectional morphology before and after moisture absorption was observed at magnifications of 2000× and 10,000×, respectively, using Field Emission Scanning Electron Microscope (Quantum SEM 5000, CIQTEK, Hefei, China).

### 4.5. Study on Water Diffusion of the Soft Capsules

#### 4.5.1. Determination of the Rate of Mass Change of Soft Capsules

The three saturated salt solutions (NaBr, NaCl, KNO_3_) were selected to control the relative humidity at different temperatures (30 °C, 35 °C and 40 °C). The soft capsules were placed in the above conditions. Then, the soft capsules were removed at 0, 0.083, 0.25, 0.5, 1, 1.5, 2, 3, 4, 6, 8, 10, 12, 16, 20, 24, 32, 36 and 48 h. After 48 h, the samples were weighed every 12 h until moisture absorption equilibrium was reached. The soft capsules were immediately weighed using an electronic balance. The moisture absorption was determined by the rate of mass change:

(5) $$W_{t}=\frac{M_{t}-M_{0}}{M_{0}}\times 100\%$$

where *W_t_* is the rate of mass change at time t; *M_t_* is the weight of the wet specimen at time t; *M*_0_ is the initial weight of the soft capsules.

#### 4.5.2. The Establishment of Fickian Diffusion Model of Soft Capsules

The diffusion behavior of soft capsules can be determined by the water transport dynamic equation, as shown in Equation (6) [28]. Fickian diffusion behavior was identified by the release depending on the initial t^1/2^ time, with Fickian diffusion defined as n = 0.50 and non-Fickian diffusion defined as n > 0.50.

(6) $$\frac{W_{t}}{W_{\infty }}=kt^{n}$$

where *W_t_* is the rate of mass change at time t, *W_∞_* is the equilibrium water content, and *k* and *n* are diffusion constants.

When $\frac{W_{t}}{W_{\infty }}\leq 0.6$, the initial part of the curve can be correlated by

(7) $$\frac{W_{t}}{W_{\infty }}=\frac{4}{L}\sqrt{\frac{D\times t}{\pi }}$$

where *D* is the diffusion coefficient and L is the thickness of the soft capsule shells.

By multiplying both sides of Equation (7) by *W_∞_*, Equation (8) was obtained.

(8) $$W_{t}=\frac{4W_{\infty }\sqrt{D}}{\sqrt{\pi }}\times \frac{\sqrt{t}}{L}=k’\frac{\sqrt{t}}{L}$$

In Equation (8), k′ represents $\frac{4W_{\infty }\sqrt{D}}{\sqrt{\pi }}$.

#### 4.5.3. The Fitting of Arrhenius Formula of Soft Capsules

The Arrhenius equation describes the relationship between k′ and temperature [29]:

(9) $$k^{l}=D_{0}\;\exp(-\frac{E_{D}}{RK})$$

where *D*_0_ was diffusion constant, which directly represents the moisture absorption capacity of the material. *E_D_* was the activation diffusion energy, which represents the minimum energy barrier for diffusion. *R* was gas constant (8.31 J·mol^−1^·K^−1^) and *T* was the absolute temperature in Kelvin. Equation (10) was obtained by taking the natural logarithm of both sides of Equation (9):

(10) $$\ln(k’)=\ln D_{0}\;-\;\frac{E_{D}}{RK}$$

### 4.6. Research on Mechanical Damage Behavior of Soft Capsules

#### 4.6.1. Determination of Mechanical Properties of Soft Capsules

The two saturated salt solutions (NaCl, KNO_3_) were selected to control the relative humidity at different temperatures (35 °C, 40 °C). The soft capsules were placed at the above conditions. At the same time points specified in Section 4.5.1, soft capsules were removed from the desiccator and immediately subjected to puncture and tensile tests.

In the puncture test, the extracted soft capsule shell was secured in the TA-FSF fixture. A puncture probe with a diameter of 1 mm was used to conduct the test at a speed of 1.5 mm/s until the shell was punctured. Based on the resulting load–distance curve, the peak value of the curve was recorded as the puncture load (g). Each experiment was performed three times, and the average value was taken.

In the tensile test, capsule rings (width: 4 mm) were fixed in the TA-CLT fixture. The fixture was operated at a speed of 1.5 mm/s until the capsule shell fractured. Based on the resulting load–distance curve, the peak value of the curve was recorded as the tensile load (g). Each experiment was conducted three times, and the average value was taken.

#### 4.6.2. Fitting Formula for the Variation in Mechanical Properties of Soft Capsules

According to the mechanical failure curves, it can be observed that the mechanical properties initially decreased rapidly over time, then more slowly, and finally stabilized. Therefore, Equation (11) was chosen to describe the variation in the mechanical indicators:

(11) $$\frac{P}{P_{0}}=\frac{1}{(t+A{)}^{K}}+B$$

where *P* is the mechanical properties of the soft capsule at time t; *P*_0_ is the mechanical properties at initial time; *A*, *B*, and *K* are constants that depend on the material characteristics, shape function, and condition.

According to the changing trend of the fitting curves, the tangents to the inflection points of the curve were drawn, and the abscissa of the intersection point of the two tangents was the shelf life.

#### 4.6.3. Prediction of Shelf Life of Soft Capsules

This study was conducted to extrapolate the shelf life of Huoxiang Zhengqi Soft Capsules under normal storage conditions using experimental data from relatively harsh environments. Because the shelf life was influenced by two key factors, temperature and humidity, and there was a synergistic effect between two variables, it was necessary to select a kinetic model capable of describing the interactive effects of multiple variables. Thus, the generalized Eyring model was selected [30]:

(12) $$\xi =\frac{A}{T}e^{\frac{B}{KT}}e^{H(C+\frac{D}{KT})}$$

where *H* was the relative humidity; *T* was the absolute storage temperature (K); *K* was the Boltzmann constant (1.381 × 10^−23^ J/K); ξ was the shelf life; *A*, *B*, *C*, and *D* were constants. Equation (13) was obtained by taking the natural logarithm of both sides of Equation (12):

(13) $$\ln\xi T=a+b\varphi_{1}(T)+c\varphi_{2}(H)+d\varphi_{1}(T)\varphi_{2}(H)$$

where a = ln A, b = B/k, c = C, d = D/k, φ_1_ (T) = 1/T, φ_2_ (H) = H.

#### 4.6.4. Statistical Analysis

Non-linear and linear regression analyses were done using OriginPro 2019b. The adequacy of the regression was evaluated using the coefficient of determination (*R*^2^).

## Figures and Tables

**Table 1:** The fitting results of the GAB model at different temperatures.

Temperature	X_0_	K	C	R^2^
30 °C	3.79	0.99	7.02 × 10^45^	0.894
35 °C	2.45	1.02	9.61 × 10^45^	0.948
40 °C	3.49	0.97	2.56 × 10^45^	0.934

**Table 2:** Diffusion kinetic parameters of the films under different environments.

Temperature	Humidity	k	*n*	R^2^
30 °C	NaBr	0.02	0.45	0.923
	NaCl	0.97	0.46	0.973
	KNO_3_	3.60	0.50	0.997
35 °C	NaBr	0.25	0.40	0.947
	NaCl	1.78	0.47	0.964
	KNO_3_	2.68	0.59	0.994
40 °C	NaBr	0.08	0.40	0.986
	NaCl	1.63	0.60	0.967
	KNO_3_	2.51	0.41	0.973

**Table 3:** Initial moisture absorption coefficient of the soft capsules.

Temperature	Humidity Environment	k′	R^2^
30 °C	NaBr	0.07014	0.985
	NaCl	0.49830	0.972
	KNO_3_	1.55351	0.997
35 °C	NaBr	0.12706	0.965
	NaCl	0.92105	0.977
	KNO_3_	1.88073	0.961
40 °C	NaBr	0.04878	0.988
	NaCl	1.61549	0.926
	KNO_3_	2.20363	0.999

**Table 4:** Changes in puncture load of the soft capsules with time.

Experiment	Experimental Conditions		R^2^	Failure Time (h)
Puncture test	35 °C, 74.87% RH	$P=\frac{2158.06}{{\left(t+0.8947\right)}^{0.3888}}-85.5455$	0.977	5.39
	35 °C, 90.79% RH	$P=\frac{2158.06}{{\left(t+0.7971\right)}^{0.4717}}-301.783$	0.968	2.41
	40 °C, 74.68% RH	$P=\frac{1946.86}{{\left(t+0.8139\right)}^{0.475}}-103.9233$	0.942	2.12
	40 °C, 89.03% RH	$P=\frac{1946.86}{{\left(t+1.0783\right)}^{3.472}}-434.7533$	0.985	0.46
Tensile test	35 °C, 74.87% RH	$P=\frac{2549.25}{{\left(t+1.2941\right)}^{1.0413}}+266.193$	0.967	4.06
	35 °C, 90.79% RH	$P=\frac{2549.25}{{\left(t+1.0407\right)}^{2.8395}}+158.6398$	0.961	1.31
	40 °C, 74.68% RH	$P=\frac{2096.00}{{\left(t+1.1254\right)}^{2.3568}}+315.7624$	0.946	1.39
	40 °C, 89.03% RH	$P=\frac{2096.00}{{\left(t+1.0121\right)}^{5.4423}}+65.2275$	0.974	0.32

**Table 5:** Relative humidity of solution.

		30 °C	35 °C	40 °C
CH_3_COOK	RH	21.61%	23.6%	25.8%
	A_w_	0.22	0.24	0.26
MgCl_2_	RH	32.44%	32.05%	31.60%
	A_w_	0.32	0.32	0.32
K_2_CO_3_	RH	43.17%	43.19%	45.51%
	A_w_	0.43	0.43	0.46
NaBr	RH	56.03%	54.55%	53.17%
	A_w_	0.56	0.54	0.53
NaCl	RH	75.09%	74.87%	74.68%
	A_w_	0.75	0.75	0.75
KNO_3_	RH	92.31%	90.79%	89.03%
	A_w_	0.92	0.91	0.89
H_2_O	RH	94.97%	95.46%	99.3%
	A_w_	0.95	0.95	0.99

## Data Availability

The data presented in this study are available on request from the corresponding author.
